# Supplementary material for: Reversible Power-to-Gas systems for energy conversion and storage
Source: Nat Commun. 2022 Apr 19;13:2010. doi: 10.1038/s41467-022-29520-0 (PMC9019040; doi:10.1038/s41467-022-29520-0)
Supplement: Supplementary file 1 — Supplementary Information [file 41467_2022_29520_MOESM1_ESM.pdf]

Supplementary Information to "Reversible Power-to-Gas Systems for Energy Conversion and Storage"

Gunther Glenk, University of Mannheim and Massachusetts Institute of Technology,
Stefan Reichelstein, University of Mannheim, Stanford University,
and Leibniz Centre for European Economic Research (ZEW)

Supplementary Table 1. List of symbols and acronyms.

α	Effective corporate income tax rate	kW	Kilowatt
c	Cost of capacity per hour	kWh	Kilowatt hour
$CF(t)$	Capacity factor at time t	L	Levelization factor
CFL_i	After-tax cash flow in year i	LFC	Levelized fixed cost
$CM(t)$	Contribution margin at time t	m	Number of hours per year
Δ	Tax factor	$\mu(t)$	Deviation factor of prices
d_i	Allowable tax depreciation in year i	p	Hydrogen price
$\epsilon(t)$	Deviation factor of generation	PEM	Polymer electrolyte membrane
η_h	Conversion rate from electricity to hydrogen	PtG	Power-to-Gas
η_e	Conversion rate from hydrogen to electricity	$q(t)$	Electricity price at time t
f	Fixed operating cost per hour	r	Cost of capital
F_i	Fixed operating cost in year i	SOC	Solide oxide cell
γ	Discount factor	t	Hour within year i
Γ	Co-variation coefficient	T	Useful life of capacity investment
GtP	Gas-to-Power	v	System price of capacity
I_i	Taxable income in year i	w	Variable cost markup per kWh
kg	Kilogram	x	Annual degradation rate of capacity

Supplementary Table 2. Input variables for modular reversible Power-to-Gas.

Input Variable	Germany	Texas	Source
**Electrolysis**			
System price, v_h^o	1,606 €/kW	1,799 \$/kW	Ref.[1]
Fixed operating cost, F_{hi}^o	48.18 €/kW	53.96 \$/kW	Ref.[1]
Conversion rate to hydrogen, η_h^o	0.019 kg/kWh	0.019 kg/kWh	Ref.[1]
**Gas Turbine**			
System price, v_e^o	1,000 €/kW	1,199 \$/kW	Ref.[2]
Fixed operating cost, F_{ei}^o	20.00 €/kW	22.40 \$/kW	Ref.[2]
Conversion rate to electricity, η_e^o	20.00 kWh/kg	20.00 kWh/kg	Ref.[2]
**Either subsystem**			
Economic lifetime, T^o	25 years	25 years	Ref.[3]
Corporate income tax rate, α^o	30.00%	21.00%	German and U.S. Tax Code
Degradation rate, x^o	0.08%	0.08%	Ref.[4]
Depreciation rate, d_i^o	6.25% (16y linear)	100% Bonus	Ref.[5;6]
Cost of capital, r^o	4.00%	6.00%	Ref.[7;8]
Electricity market price (2019), q	3.77 €¢/kWh	3.77 \$¢/kWh	`www.eex.com`; `www.ercot.com`
Cost markup for electricity generation, w_e^o	0.00 €¢/kWh	0.00 \$¢/kWh	Hydrogen price includes supply
Cost markup for hydrogen generation, w_h^o	0.38 €¢/kWh	1.00 \$¢/kWh	See Supplementary Table 4–5

Supplementary Table 3. Input variables for integrated reversible Power-to-Gas.

Input Variable	Germany	Texas	Source
System price, v	2,243 €/kW	2,512 $/kW	Own review, see Methods
Fixed operating cost, F	67.29 €/kW	75.36 $/kW	Own review, see Methods
Conversion rate to hydrogen, η_h	0.023 kg/kWh	0.023 kg/kWh	Ref. [2;9–11]
Conversion rate to electricity, η_e	20.00 kWh/kg	20.00 kWh/kg	Ref. [2;9;12;13]
Economic lifetime, T	15 years	15 years	Ref. [13]
Corporate income tax rate, α	30.00%	21.00%	German and U.S. Tax Code
Degradation rate, x	1.60%	1.60%	Ref. [12]
Depreciation rate, d_i	6.67% (15y linear)	100% Bonus	Ref. [5;6]
Cost of capital, r	4.00%	6.00%	Ref. [7;8]
Electricity market price (2019), q	3.77 €¢/kWh	3.77 $¢/kWh	`www.eex.com`; `www.ercot.com`
Cost markup for electricity generation, w_e	0.00 €¢/kWh	0.00 $¢/kWh	Hydrogen price includes supply
Cost markup for hydrogen generation, w_h	0.42 €¢/kWh	1.05 $¢/kWh	See Supplementary Table 4–5

Supplementary Table 4. Cost markup for hydrogen generation, Germany

Variable	Value	Source
**Electricity price markup**		
Transmission charge (€¢/kWh)	0.000	§118 (6) Energiewirtschaftsgesetz
Concession charge (€¢/kWh)	0.000	§118 (6) Energiewirtschaftsgesetz
EEG levy (€¢/kWh)	0.100	§64 (2) with A. 4 Erneuerbare-Energien-Gesetz
CHP levy (€¢/kWh)	0.030	§27 (1) Kraft-Wärme-Kopplungsgesetz
§19 StromNEV levy (€¢/kWh)	0.025	§19 (2) Stromnetzentgeltverordnung
Offshore liability levy (€¢/kWh)	0.030	§17f (5) Energiewirtschaftsgesetz
Levy for interruptable loads (€¢/kWh)	0.000	§18 Verordnung zu abschaltbaren Lasten
Electricity tax (€¢/kWh)	0.000	§9a (1) 1. Stromsteuergesetz
Total electricity price markup (€¢/kWh)	0.185	
**Other variable cost**		
Cost for water and other consumables (€/kg)	0.100	Estimation

Supplementary Table 5. Cost markup for hydrogen generation, Texas

Variable	Value	Source
**Electricity price markup**		
Transmission and distribution charges ($¢/kWh)	0.0077	Ref. [14], transmission rate
Transmission system charge ($¢/kWh)	0.3055	Ref. [14], transmission rate
Distribution system charge ($¢/kWh)	0.0668	Ref. [14], transmission rate
System benefit fund charge ($¢/kWh)	0.0000	Ref. [14], Rider SBF
Transition charge ($¢/kWh)	0.0000	Ref. [14], Schedules TC
Nuclear decommissioning charge ($¢/kWh)	0.0001	Ref. [14], Rider NDC
Transmission cost recovery factor ($¢/kWh)	0.3094	Ref. [14], Rider TCRF
Competition transition charge ($¢/kWh)	0.0000	Ref. [14], Rider CTC
Competitive metering credit ($¢/kWh)	0.0001	Ref. [14], Rider CMC
Other charges or credits ($¢/kWh)	0.0731	Ref. [14], Riders RCE, EECRF, DCRF
Griddy membership fee ($¢/kWh)	0.0061	Ref. [15]
Taxes ($¢/kWh)	0.0192	U.S. Tax Code
Total electricity price markup ($¢/kWh)	0.7880	
**Other variable cost**		
Cost for water and other consumables ($/kg)	0.1120	Conversion of € value to $ with avg. exchange rate of 2019

Supplementary Table 6. Current economics of modular reversible Power-to-Gas.

	Germany	Texas
**Power-to-Gas Subsystem**		
Contribution margin of hydrogen $CM_h^o(p_h^o)$	1.15 €¢/kWh	2.39 $¢/kWh
Break-even price for hydrogen, p_h^o	3.18 €/kg	2.98 $/kg
Capacity factor for hydrogen, $CF_h^o(p_h^o)$	0.94	0.93
Levelized fixed cost, LFC_h^o	2.01 €¢/kWh	2.39 $¢/kWh
Levelized fixed operating cost, f_h^o	0.60 €¢/kWh	0.66 $¢/kWh
Levelized capacity cost, c_h^o	1.27 €¢/kWh	1.73 $¢/kWh
Tax factor, Δ_h^o	1.12	1.00
**Gas-to-Power Subsystem**		
Contribution margin of electricity, $CM_e^o(p_e^o)$	1.15 €¢/kWh	1.35 $¢/kWh
Break-even price for hydrogen, p_e^o	0.57 €/kg	1.31 $/kg
Capacity factor for electricity, $CF_e^o(p_e^o)$	0.82	0.04
Levelized fixed cost, LFC_e^o	1.13 €¢/kWh	1.35 $¢/kWh
Levelized fixed operating cost, f_e^o	0.25 €¢/kWh	0.27 $¢/kWh
Levelized capacity cost, c_e^o	0.79 €¢/kWh	1.07 $¢/kWh
Tax factor, Δ_e^o	1.12	1.00

Supplementary Table 7. Current economics of integrated reversible Power-to-Gas.

	Germany	Texas
**Upper Break-even Price**		
Contribution margin of hydrogen $CM_h(p^*)$	3.68 €¢/kWh	3.04 $¢/kWh
Break-even price for hydrogen, p^*	3.38 €/kg	2.78 $/kg
Capacity factor for hydrogen, $CF_h(p^*)$	0.99	0.95
Contribution margin of electricity, $CM_e(p^*)$	0.00 €¢/kWh	1.15 $¢/kWh
Capacity factor for electricity, $CF_e(p^*)$	0.00	0.02
**Lower Break-even Price**		
Contribution margin of hydrogen $CM_h(p_*)$	0.03 €¢/kWh	0.00 $¢/kWh
Break-even price for hydrogen, p_*	0.03 €/kg	-0.09 $/kg
Capacity factor for hydrogen, $CF_h(p_*)$	0.02	0.00
Contribution margin of electricity, $CM_e(p_*)$	3.66 €¢/kWh	4.22 $¢/kWh
Capacity factor for electricity, $CF_e(p_*)$	0.97	1.00
**Either Break-even Price**		
Levelized fixed cost, LFC	3.67 €¢/kWh	4.19 $¢/kWh
Levelized fixed operating cost, f	0.85 €¢/kWh	0.94 $¢/kWh
Levelized capacity cost, c	2.54 €¢/kWh	3.24 $¢/kWh
Tax factor, Δ	1.11	1.00

Supplementary Table 8. Prospects, Germany.

Year	v_h^o (€/kW)	η_h^o (kWh/kg)	p_h^o (€/kg)	p_e^o (€/kg)	v (€/kW)	η_h (kWh/kg)	η_e (kWh/kg)	p^* (€/kg)	p_* (€/kg)	$\bar{p}$ (€/kg)	$\underline{p}$ (€/kg)
2019	1,606	0.019	3.18	0.57	2,243	0.023	20.00	3.38	0.03	2.43	-1.81
2020	1,530	0.020	3.08	0.57	2,042	0.023	20.15	3.23	0.10	2.45	-1.82
2021	1,457	0.020	2.99	0.57	1,859	0.023	20.30	3.09	0.16	2.47	-1.83
2022	1,387	0.020	2.90	0.57	1,693	0.023	20.45	2.96	0.23	2.49	-1.85
2023	1,321	0.020	2.81	0.57	1,541	0.024	20.61	2.84	0.28	2.51	-1.86
2024	1,258	0.021	2.73	0.57	1,403	0.024	20.76	2.73	0.34	2.53	-1.87
2025	1,198	0.021	2.65	0.57	1,278	0.024	20.91	2.63	0.39	2.54	-1.89
2026	1,141	0.021	2.58	0.57	1,163	0.024	21.06	2.54	0.43	2.56	-1.90
2027	1,086	0.022	2.51	0.57	1,059	0.024	21.21	2.45	0.48	2.58	-1.91
2028	1,035	0.022	2.44	0.57	964	0.024	21.36	2.37	0.52	2.60	-1.93
2029	985	0.022	2.37	0.57	878	0.024	21.52	2.30	0.55	2.62	-1.94
2030	938	0.023	2.31	0.57	799	0.024	21.67	2.23	0.59	2.64	-1.96

Supplementary Table 9. Prospects, Texas.

Year	v_h^o (\$/kW)	η_h^o (kWh/kg)	p_h^o (\$/kg)	p_e^o (\$/kg)	v (\$/kW)	η_h (kWh/kg)	η_e (kWh/kg)	p^* (\$/kg)	p_* (\$/kg)	$\bar{p}$ (\$/kg)	$\underline{p}$ (\$/kg)
2019	1,799	0.019	2.98	1.31	2,512	0.023	20.00	2.78	-0.09	>5.00	0.59
2020	1,713	0.020	2.88	1.31	2,287	0.023	20.15	2.59	-0.01	>5.00	0.58
2021	1,631	0.020	2.77	1.31	2,082	0.023	20.30	2.42	0.06	>5.00	0.58
2022	1,553	0.020	2.68	1.31	1,896	0.023	20.45	2.26	0.12	>5.00	0.58
2023	1,479	0.020	2.59	1.31	1,726	0.024	20.61	2.11	0.18	>5.00	0.58
2024	1,409	0.021	2.51	1.31	1,572	0.024	20.76	1.97	0.24	>5.00	0.58
2025	1,342	0.021	2.42	1.31	1,431	0.024	20.91	1.84	0.29	>5.00	0.58
2026	1,278	0.021	2.35	1.31	1,303	0.024	21.06	1.72	0.35	>5.00	0.57
2027	1,217	0.022	2.28	1.31	1,186	0.024	21.21	1.60	0.41	>5.00	0.57
2028	1,159	0.022	2.21	1.31	1,080	0.024	21.36	1.48	0.50	>5.00	0.57
2029	1,103	0.022	2.14	1.31	983	0.024	21.52	1.35	0.64	>5.00	0.57
2030	1,051	0.023	2.08	1.31	895	0.024	21.67	1.17	0.89	>5.00	0.57

Supplementary Note 1. Heating Costs

To account for the cost of heating, we again adopt the framework of hourly operating decisions that underlies all our calculations. Thus, in the following t is an integer with $1 \leq t \leq 8,760$ hours. For an integrated reversible PtG system, suppose that after it has been idle for a particular period of time, it takes the system $\Delta \cdot 60$ minutes to heat up to regular operating temperature after a cold start. During this heat-up phase, suppose the system incurs the full variable cost at full capacity utilization and, at the same time, obtains no output yet. In this arguably conservative representation, we then obtain the following "penalty" factor $\theta(t)$ that the system incurs during a heat-up phase:

$$\theta(t) = \begin{cases} 1 - \Delta & \text{if } CF_h(t-1|p) = CF_e(t-1|p) = 0, \\ 1 & \text{otherwise.} \end{cases}$$

The corresponding contribution margins then are:

$$CM_h(CF_h, t|p) = \big[\eta_h(CF_h) \cdot p \cdot \theta(t) - q(t) - w_h\big] \cdot CF_h,$$

for hydrogen production, and

$$CM_e(CF_e, t|p) = \big[q(t) \cdot \theta(t) - \frac{p}{\eta_e(CF_e)} - w_e\big] \cdot CF_e,$$

for electricity. For modular reversible PtG systems, the corresponding equations are derived in direct analogy to the approach shown above. For a gas turbine, the cost of heating can be interpreted as the cost of a cold start-up.

Note that the above specifications of the contribution margins constitute upper bounds, considering that electrolyzer and fuel cell technologies based on SOC or PEM can produce some output during heat-up yet at a diminished conversion efficiency[4;16]. Similarly, gas turbines can produce some output during the ramp-up to full capacity utilization after a cold start[17]. Furthermore, a high-temperature SOC electrolyzer requires only a small fraction of the energy input needed for the subsequent electrolytic hydrogen production in order to reach operating temperature[18].

We then evaluate the modified model framework in the same economic context as in the main body. The heat-up to operating temperature of either a modular or an integrated PtG system based on PEM (SOC) technology requires less than 10 (20) minutes[4;19]. We thus assume conservatively that $\Delta = \frac{1}{6}$ for the modular electrolyzer and $\Delta = \frac{1}{3}$ for an integrated system. Since cold start-up times for combined-cycle gas turbines can be below 60 minutes[17], let here $\Delta = 1$. Furthermore, suppose conservatively that either reversible PtG equipment has to heat up (start up) whenever it has been idle for at least 1 hour.

The resulting break-even values in the current economic context are summarized in Supplementary Table 10. For the modular electrolyzer and the integrated system, these break-even values are close to those shown in Table 2 of the main body. For instance, for the integrated PtG system in Texas the upper and lower break-even prices in Supplementary Table 10 amount to \$2.81/kg and \$-0.09/kg, respectively, while the corresponding prices in Table 2 of the main body amount to \$2.78/kg and \$-0.09/kg, respectively. The break-even price for the modular gas turbine in Texas is about 40% lower if heating costs are included. This relatively large effect stems from the fact that, given the assumptions made, the gas turbine must perform multiple cold starts per year. The modular electrolyzer and the integrated system, in contrast, operate for most of the time and must go only through a few heat-up phases per year.

Supplementary Table 10. Current economics including heating cost.

	Germany	Texas
**Modular Reversible PtG System**		
Break-even price of Power-to-Gas: p_h^o	3.18 €/kg	2.98 \$/kg
Break-even price of Gas-to-Power: p_e^o	0.55 €/kg	0.79 \$/kg
**Integrated Reversible PtG System**		
Upper break-even price: p^*	3.38 €/kg	2.81 \$/kg
Lower break-even price: p_*	0.03 €/kg	-0.09 \$/kg
Upper critical price: $\bar{p}$	1.99 €/kg	>5.0 \$/kg
Lower critical price: $\underline{p}$	-1.81 €/kg	0.80 \$/kg

Supplementary Note 2. Sensitivity to Conversion Efficiencies

We examine the sensitivity of our numerical findings for integrated reversible PtG systems based on SOC technology to changes in the conversion efficiencies in two regards. First, we examine the possibility that the conversion rates at full capacity utilization are higher or lower than the values we identified in the literature. For that, we run the corresponding calculations of the main body for both jurisdictions assuming for brevity that each conversion rate is adjusted by the same factor $\Delta \in [-20\%, +20\%]$. The resulting %-change in the relevant prices for hydrogen is summarized in Supplementary Figure 1.

Figure 1 shows that the insights obtained from the main body of the paper are generally robust to the relatively wide range of changes in the conversion rates. The lower break-even price for hydrogen of an integrated system in either Germany or Texas is unaffected by a change in conversion efficiencies. The lower critical hydrogen price of an integrated system in Germany is upward sloping, because the underlying hydrogen price is negative. In absolute terms, the lower critical hydrogen price declines as the conversion efficiency increases.

In the second sensitivity analysis, we examine the trade-off between full and partial capacity utilization that could arise if the conversion rates of a unitized regenerative SOC fuel cell were to increase significantly at capacity utilization rates of less than 100%. Given the lack of evidence regarding the shape of the functions $\eta_h(\cdot)$ and $\eta_e(\cdot)$, we first examine, in particular, the increase in both conversion rates that must be obtained if the integrated reversible PtG system operated at or below a particular capacity utilization of less than 100% such that the upper break-even price for hydrogen remains unchanged. We use the upper break-even price as the measure of indifference, because the integrated PtG system mostly produces hydrogen.

If hypothetically the integrated system were to achieve its maximum conversion rates at 70% capacity utilization, we find that the two conversion rates would each have to increase by 20.8% in Germany and 29.0% in Texas for the upper break-even price for hydrogen to remain

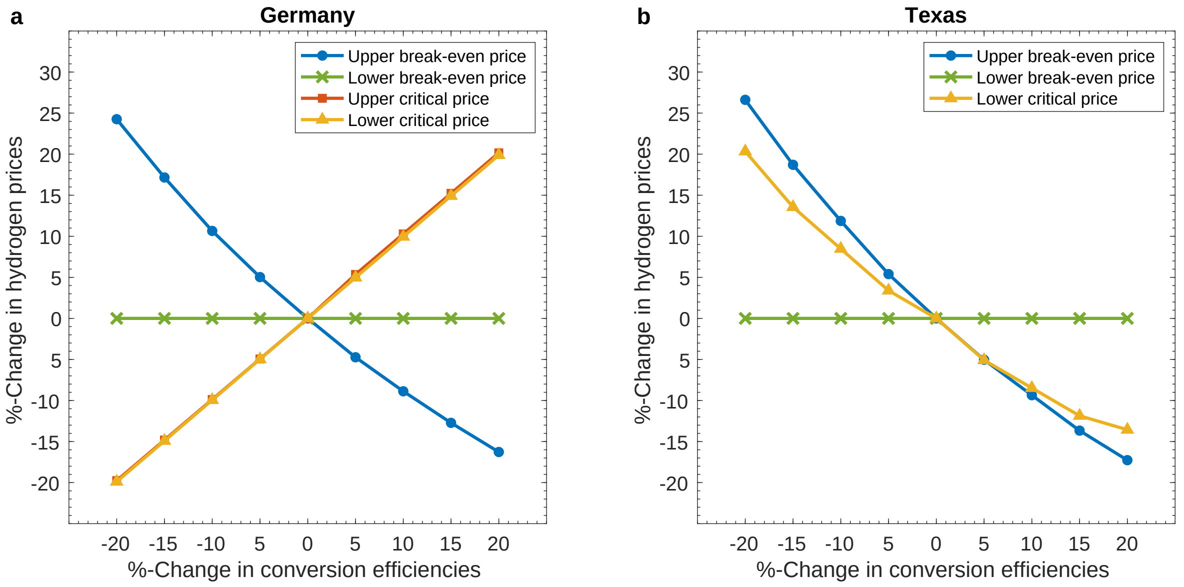

Supplementary Figure 1. Sensitivity to alternative conversion efficiencies. a,b, This figure shows the %-change in break-even prices for hydrogen of integrated reversible Power-to-Gas systems in (**a**) Germany and (**b**) Texas as a function of the same %-change in both conversion efficiencies of the system. The upper critical price for the integrated system in Texas is consistently above 5.0\$/kg and therefore omitted.

unchanged. Thus, if for the system in Texas $\eta_h(CF_h = 0.7)$ and $\eta_e(CF_e = 0.7)$ is 1.29 times the corresponding values at full capacity utilization, the system would attain the same competitive position by operating at full capacity utilization despite the lower conversion rates. This analysis shows that the two conversion rates would have to decrease steeply for capacity utilization values approaching one for the integrated system to obtain a higher net present value by operating at partial load.

To further explore the magnitude of such a potential trade-off, let $\eta_h(CF_h = 0.7)$ and $\eta_e(CF_e = 0.7)$ hypothetically each amount to values that are as much as 35% above those at full capacity utilization. The resulting current upper break-even price for the integrated system for the scenario calculated in the main body then amounts to 3.03€/kg in Germany and 2.66 \$/kg in Texas. These values are about 10% and 4% lower than the respective upper break-even prices obtained for the scenario calculated in the main body. This analysis shows that even if conversion rates at a 70% utilization increased as steeply as 1.35 times the corresponding values at full capacity utilization, the overall effect on the competitive position of the integrated system is relatively minor. The reason is that the increase in conversion efficiencies is partially offset by the falling capacity utilization.

Supplementary Note 3. Real-time Market Prices for Electricity

We also run our calculations using hourly prices for electricity from 2019 for the real-time wholesale market in Texas. The resulting break-even prices for the current economic environment are shown in Supplementary Table 11. As it can be seen, differences of corresponding break-even values between day-ahead and real-time market prices for electricity are small if observable at all. Break-even prices for modular gas turbines are now borderline to hydrogen prices for industrial-scale supply.

Supplementary Table 11. Current economics based on real-time prices for Texas.

	**Texas**
**Modular reversible PtG System**	
Break-even price of Power-to-Gas: p_h^o	2.98 \$/kg
Break-even price of Gas-to-Power: p_e^o	1.53 \$/kg
**Integrated reversible PtG System**	
Upper break-even price: p^*	2.76 \$/kg
Lower break-even price: p_*	-0.08 \$/kg
Upper critical price: $\bar{p}$	>5.0 \$/kg
Lower critical price: $\underline{p}$	-0.03 \$/kg

Supplementary References

[1] Glenk, G. & Reichelstein, S. Economics of converting renewable power to hydrogen. *Nature Energy* **4**, 216–222 (2019).

[2] IEA. The Future of Hydrogen. Tech. Rep. (2019).

[3] Michalski, J. *et al.* Hydrogen generation by electrolysis and storage in salt caverns: Potentials, economics and systems aspects with regard to the German energy transition. *International Journal of Hydrogen Energy* **42**, 13427–13443 (2017).

[4] Buttler, A. & Spliethoff, H. Current status of water electrolysis for energy storage, grid balancing and sector coupling via power-to-gas and power-to-liquids: A review. *Renewable and Sustainable Energy Reviews* **82**, 2440–2454 (2018).

[5] Bundesfinanzhof. BFH-Urteil 14.04.2011 IV R 52/10 (Bundesfinanzhof, 2011).

[6] U.S. Congress. H.R.1: An Act to provide for reconciliation pursuant to titles II and V of the concurrent resolution on the budget for fiscal year 2018. (2017).

[7] Fraunhofer ISI. The impact of risks in renewable energy investments and the role of smart policies. Tech. Rep. (2016).

[8] Moné, C., Stehly, T., Maples, B. & Settle, E. 2014 Cost of Wind Energy Review. Tech. Rep. February (2015).

[9] Weidner, E., Ortiz Cebolla, R. & Davies, J. Global deployment of large capacity stationary fuel cells. Tech. Rep. (2019).

[10] SunFire GmbH. Technology details of our SOEC. Tech. Rep. (2018).

[11] Peterson, D. & Miller, E. Hydrogen production cost from solid oxide electrolysis. Tech. Rep. (2016).

[12] U.S. Department of Energy. Report on the Status of the Solid Oxide Fuel Cell Program. Tech. Rep. (2019).

[13] Elcogen. Solid Oxide Fuel Cells: Opportunities for a clean energy future. Tech. Rep. (2019).

[14] CenterPoint Energy Houston Electric. Tariff for Retail Delivery Service (2020). URL `http://bit.ly/382OFJO`.

[15] Griddy. Pricing (2020). URL http://bit.ly/382npZk.

[16] Mogensen, M. B. *et al.* Reversible solid-oxide cells for clean and sustainable energy. *Clean Energy* **3**, 175–201 (2019).

[17] U.S. EIA. About 25% of U.S. power plants can start up within an hour. 07.06.2021 (2020). URL https://www.eia.gov/todayinenergy/detail.php?id=45956.

[18] Graves, C., Ebbesen, S. D., Mogensen, M. & Lackner, K. S. Sustainable hydrocarbon fuels by recycling CO2 and H2O with renewable or nuclear energy. *Renewable and Sustainable Energy Reviews* **15**, 1–23 (2011).

[19] Beney, A. M. Investigation into the heat up time for solid oxide fuel cells in automotive applications. Tech. Rep. (2018).
