# Supplementary material for: Reversible Power-to-Gas systems for energy conversion and storage
Source: Nat Commun. 2022 Apr 19;13:2010. doi: 10.1038/s41467-022-29520-0 (PMC9019040; doi:10.1038/s41467-022-29520-0)
Supplement: Supplementary file 3 — Description of Additional Supplementary Files [file 41467_2022_29520_MOESM3_ESM.pdf]

Description of Additional Supplementary Files

File Name: Supplementary Data 1
Description: The supplementary data documents the review and the data set of the cost dynamics of solid oxide cells.
